# The Effect of the Thioether-Bridged, Stabilized Angiotensin-(1–7) Analogue Cyclic Ang-(1–7) on Cardiac Remodeling and Endothelial Function in Rats with Myocardial Infarction

**DOI:** 10.1155/2012/536426

**Published:** 2011-10-29

**Authors:** Matej Durik, Richard van Veghel, Anneke Kuipers, Rick Rink, Marijke Haas Jimoh Akanbi, Gert Moll, A. H. Jan Danser, Anton J. M. Roks

**Affiliations:** ^1^Division of Vascular Medicine and Pharmacology, Department of Internal Medicine, Erasmus University Medical Center, Dr. Molewaterplein 50, 3015 GE Rotterdam, The Netherlands; ^2^LanthioPep, Nijenborgh 4, 9747 AG Groningen, The Netherlands; ^3^BiOMaDe Technology Foundation, Groningen, The Netherlands; ^4^Department of Pharmacology and Therapy, College of Health Sciences, University of Ilorin, Ilorin, Nigeria

## Abstract

Modulation of renin-angiotensin system (RAS) by angiotensin-(1–7) (Ang-(1–7)) is an attractive approach to combat the detrimental consequences of myocardial infarction (MI). However Ang-(1–7) has limited clinical potential due to its unfavorable pharmacokinetic profile. We investigated effects of a stabilized, thioether-bridged analogue of Ang-(1–7) called cyclic Ang-(1–7) in rat model of myocardial infarction. Rats underwent coronary ligation or sham surgery. Two weeks thereafter infusion with 0.24 or 2.4 **μ**g/kg/h cAng-(1–7) or saline was started for 8 weeks. Thereafter, cardiac morphometric and hemodynamic variables as wells as aortic endothelial function were measured. The average infarct size was 13.8% and was not changed by cAng-(1–7) treatment. MI increased heart weight and myocyte size, which was restored by cAng-(1–7) to sham levels. In addition, cAng-(1–7) lowered left ventricular end-diastolic pressure and improved endothelial function. The results suggest that cAng-(1–7) is a promising new agent in treatment of myocardial infarction and warrant further research.

## 1. Introduction

Myocardial infarction is a leading cause of mortality and morbidity in western society. Current intervention relies on prevention of myocardial hypertrophy and fibrosis and of thrombosis. Since these processes are partially mediated by an increase of the renin-angiotensin system (RAS) hormone; angiotensin (Ang) II, inhibition of this hormone through drugs; that decrease its production or its signaling via the Ang II type 1 (AT1) receptor; forms an important part of the applied pharmacotherapy. The ever culminating knowledge of RAS; brought about by relentless research of a vast group of scientists; has raised the awareness that there is more to achieve than with classical RAS intervention only. Possible novel intervention strategies have emerged, of which those based on stimulation of angiotensin-(1–7) (Ang-(1–7)) function as one of the most appealing [1, 2].

Angiotensin-(1–7) (Ang-(1–7)) is a hormone that in general counteracts Ang II through its own signaling pathways, which involves the Mas receptor [3]. Studies in animal models show that it has ample therapeutic potential in cardiovascular disease, in particular diseases that are featured by malignant remodeling of the heart. We showed that chronic infusion of Ang-(1–7) in rats or mice with myocardial infarction improves cardiac and endothelial function [4, 5]. The beneficial effect of Ang-(1–7) infusion after myocardial infarction relies on the versatile bioactivity of the hormone, which comprises antihypertrophic, -fibrotic, and -thrombotic function, improvement of eNOS function, blockade of Ang-II-induced ROS production, and stimulation of endothelial-progenitor-cell-mediated angiogenesis [1, 2, 5–9]. In spite of being a therapeutic prodigy, Ang-(1–7) does not offer ideal prospects for clinical use because of its pharmacokinetical and pharmacodynamical properties (as is also elaborated in [1]). Firstly, the peptide is rapidly metabolised in plasma and tissue. Second, beneficial effects of Ang-(1–7) take place at low concentrations at which Mas receptors are stimulated. At higher concentrations Ang-(1–7) becomes aspecific for receptor subtype binding, being a partial Ang II type 1 receptor agonist and an Ang II type 2 receptor agonist. Thus, overdosing might interfere with its Mas receptor-associated functions. 

To improve the pharmacological profile we have developed cyclic Ang-(1–7) (cAng-(1–7)), an Ang-(1–7) analogue in which amino acid residues 4 and 7 have been linked with a thioether bridge, thus forming a lanthionine [10]. The strategy of thioether bridging is used by bacteria to stabilize peptides, and we previously showed that enzymatically synthesized cAng-(1–7) was fully resistant against degradation by angiotensin-converting enzyme and had enhanced resistance against breakdown by other proteases. It displayed 34-fold enhanced presence in the blood circulation in Sprague-Dawley (SD) rats during continuous intravenous infusion. The thioether ring did not prevent cAng-(1–7) from agonistically interacting with the Mas receptor, the receptor of native angiotensin-(1–7). cAng-(1–7) even induced a twofold larger relaxation of precontracted SD rat aorta rings than native Ang-(1–7). Moreover, it is a specific agonist for Ang-(1–7) receptors. Therefore, cAng-(1–7) holds promise for use in cardiovascular therapy. In this study we have tested the effect of chronic cAng-(1–7) infusion on hemodynamic function after myocardial infarction in the rat.

## 2. Methods

### 2.1. Animals

Male Sprague-Dawley rats weighing 280–300 grams were obtained from Harlan (Horst, the Netherlands). Animals were put on standard rat chow and water, available ad. libitum. Housing was at room temperature with a 12 h light–12 h dark cycle. After at least one week of acclimatization in the caretaking facility, the rats were operated to induce left ventricular myocardial infarction (MI) or underwent a sham procedure.

### 2.2. Surgery to Induce MI and Surgical Procedures

Prior to surgery 0.01 mg/kg buprenorphine was given subcutaneously for postoperative analgesia, which was repeated after surgery for 2 days, 2 times daily. Operations were performed under 2.5% isoflurane in air ventilation anesthesia for which the rats were intubated. Through an opening in the left 4th intercostal space of the chest, MI was induced by ligation of the left coronary artery with a 6/0 silk suture. After induction of MI, as witnessed by bleaching of the myocardium, the chest was closed and animals were withdrawn from anesthesia. Sham-operated animals (SHAM) underwent an identical procedure, however, without tying the silk suture to close the coronary artery. MI surgery was performed in 106 animals, 8 animals were sham-operated. Perioperative mortality was 45% in the MI group.

### 2.3. Treatment with cAng-(1–7)

Two weeks after induction of MI, rats were randomly allocated to intravenous infusion of either 0.24 (low dose or low cAng-(1–7)) or 2.4 *μ*g/kg/h (high dose or high cAng-(1–7)) of cAng-(1–7) *n* = 12 for each dose), or of saline (*n* = 25) by 4-week osmotic minipumps (Alzet model 2004). Sham-operated controls (*n* = 8) received saline or high dose of cAng-(1–7) (2.4 *μ*g/kg/h). Animals were infused for 8 weeks, changing pumps at week 4. To accomplish intravenous infusion a polyethylene tube was implanted in the left jugular vein. cAng-(1–7) was made by BiOMade/LanthioPep, Groningen.

### 2.4. Measurements of Hemodynamic and Vascular Function

After 8 weeks of treatment animals were weighed (body weight: BW) and hemodynamic studies were performed under isoflurane anesthesia (2.5% in air) with a 2F catheter-based, microtip pressure transducer (Millar, Houston, Tex, USA) that was introduced into the left ventricle via intraluminal passing through the right carotid artery. Rats were anesthetized for 20 minutes before the start of the measurement.

After measurement of hemodynamic function the heart was excised for histological studies. After removal of ventricular blood; the heart was weighed to obtain total heart weight (HW). The thoracic aorta was isolated to perform functional studies. To this end the aorta was kept in Krebs solution in mmol/L: NaCl 118, KCl 4.7, CaCl_2_ 2.5, MgSO_4_ 1.2, KH_2_PO_4_ 1.2, NaHCO_3_ 25 and glucose 8.3; pH 7.4. Surrounding periaortic adipose tissue was carefully removed with small scissors. Rings of 2 mm length were cut and mounted in small wire organ baths containing Krebs at 37°C. To investigate the contribution of dilator signaling factors nitric oxide (NO) production was blocked using L-NAME (100 *μ*mol/L), and endothelium-derived hyperpolarizing factor (EDHF) was blocked with apamin (0.5 *μ*mol/L) and charybdotoxin (0.1 *μ*mol/L). Subsequently, in the absence or presence of these inhibitors, concentration-response curves were constructed to methacholine and SNP after preconstruction with phenylephrine. All chemicals were from Sigma-Aldrich, the Netherlands.

### 2.5. Histology

Midventricular slices of the heart were fixed with 4% formaldehyde, embedded in paraffin and processed for histochemical analysis. Infarct size was determined on picrosirius red/fast green-stained sections and was expressed as the percentage of scar length of the average of left ventricular internal and external circumference. Rats with all infarct sizes were included in the analysis. The cross-sectional area of the individual cells was measured on gomori-stained sections. Myocyte density was determined by assessment of the number of cells per tissue area for each slide and subsequent conversion to mm^2^. Fibrosis was measured on picrosirius red/fast green-stained sections from three randomly selected regions of the surviving myocardium.

### 2.6. Statistical Analysis

Data are presented as mean ± SEM. Statistical differences between the groups were evaluated by *t*-test or by 1-way ANOVA for hemodynamic and histological variables, using Dunnett's *t*-test or Bonferroni correction where appropriate. One-sided testing was applied in all bar graphs as the effects were in the expected direction. For testing of trend; linear regression analysis was applied. Differences in concentration-response curves to methacholine were tested by general linear model ANOVA for repeated measures. Differences were considered significant at *P* < 0.05.

## 3. Results

### 3.1. Weight and Histological Characteristics

General parameters at the end of treatment are shown in Table 1. No differences were observed in body weight between the 4 groups. 

Infarct sizes were in general small and did not differ significantly between the cAng-(1–7) and saline-treated group. Similarly, fibrosis did not differ between the groups (Table 1). 

**Figure 1:**
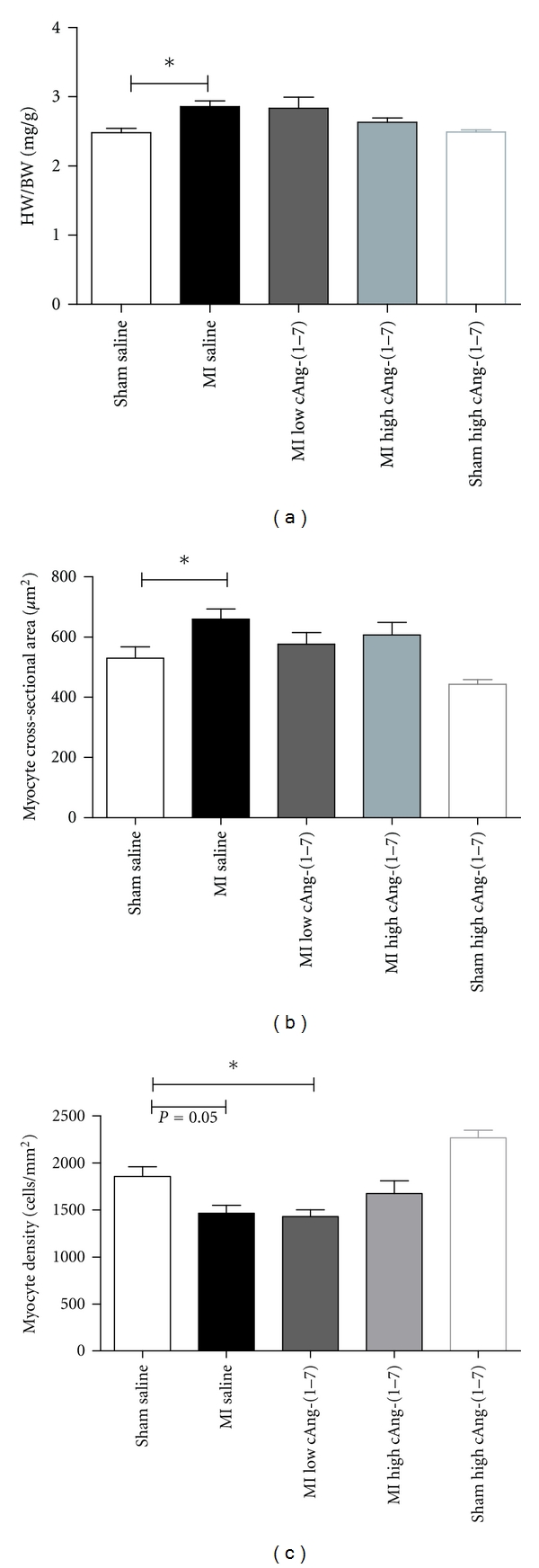
Comparison of heart weight/body weight ratios between the different treatments (a), variables of cardiac hypertrophy: myocyte, cross-sectional area (b), and myocyte density (c). (*P < 0.05, One way ANOVA, Dunnett's post hoc testing).

Despite the small infarct sizes total heart weight to body weight ratio has modestly but significantly increased in saline-treated MI group compared with SHAM (Figure 1(a)). Both doses of cAng (1–7) abolished the significant difference between MI and SHAM. However, only the higher dose of 2.4 *μ*g/kg/h cAng-(1–7) resulted in a lower heart weight compared to saline, though not significantly different. 

To further determine the cause of the weight differences the effect of cAng-(1–7) on myocyte size measured. Myocardial infarction increased myocyte cross-sectional area and decreased myocyte cell density (Figures 1(b) and 1(c)). Treatment with both doses of cAng-(1–7) restored myocyte cross-sectional area to the level of saline-treated sham (Figure 1(b)). Myocyte density was only restored by the higher dose of cAng-(1–7) (Figure 1(c)). In sham-operated animals, cAng-(1–7) treatment showed a trend towards a decrease in myocyte size, but this effect did not reach a statistical significance (Figures 1(b) and 1(c)).

### 3.2. Hemodynamics

**Figure 2:**
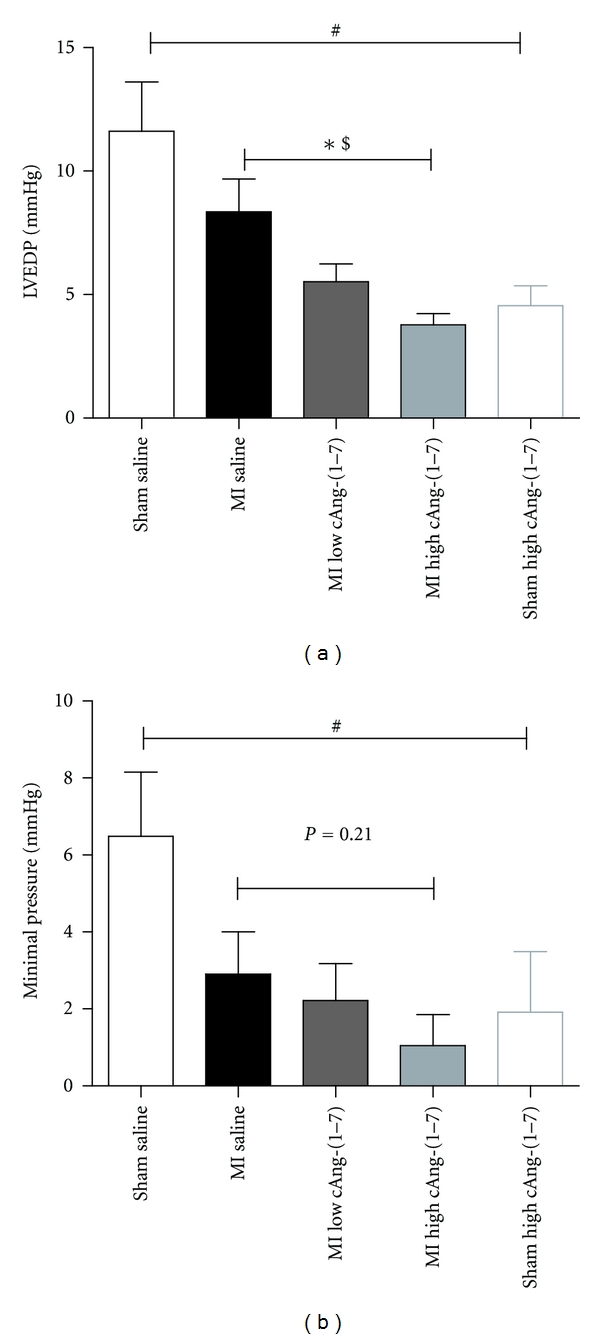
Effects of cAng 1–7 on left ventricular end-diastolic pressure and minimal pressure in both sham-operated rats and rats with myocardial infarction. (^#^ *P* < 0.05*t*-test sham saline versus sham cAng-(1–7); *P < 0.05, One way ANOVA for MI groups, Dunnett's post hoc testing; ^$^ *P* < 0.05 for linear trend for MI groups).

After 8 weeks of treatment, cardiac function was measured in vivo in anesthetized rats. In accordance with the small infarct size, cardiac function was not significantly impaired in untreated MI rats as compared with SHAM (Table 1). In agreement with the absence of systolic or diastolic heart failure MI did not significantly change left ventricular end diastolic pressure (LVEDP) or left ventricular minimal pressure (Pmin) (*t*-test, *P* = 0.199 for LVEDP; *P* = 0.090 for Pmin), and therefore the effect of cAng-(1–7) was tested within the MI and sham group, respectively (Figure 2). In the MI group, cAng(1–7) treatment lowered LVEDP which was significant at the highest doses (Figure 2(a)). Since there seemed to be a dose-dependent effect we tested for a trend line, which resulted in a significance for trend. Pmin seemed also to be lowered in MI animals, but this effect did not reach statistical significance (Figure 2(b)). In sham animals, cAng-(1–7) given at a doses of 2.4 *μ*g/kg/h lowered both LVEDP and Pmin (Figures 2(a) and 2(b)). All other measured pressure variables were not changed by cAng-(1–7) treatment as compared to MI saline (Table 1).

### 3.3. Endothelial Function

Endothelial dysfunction is a key feature in the development of heart failure after MI since it contributes to the increase of peripheral vascular resistance that leads to increased cardiac workload resulting in hypertrophy and contractile dysfunction of the myocardium. Therefore, we investigated endothelium-dependent relaxation in isolated aortic rings. 

**Figure 3:**
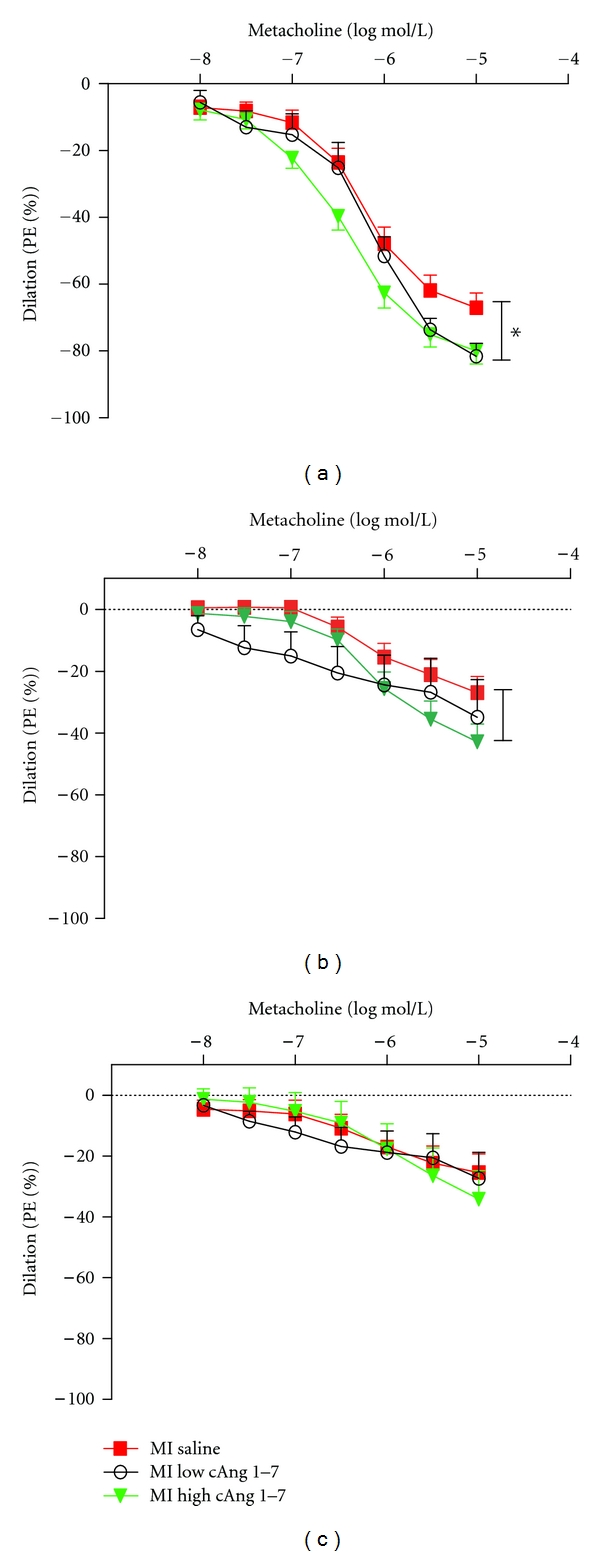
Endothelial-dependent dilator function of rat aorta to metacholine (a), after blockade of eNOS/NO signaling (b), and after combined blockade of eNOS/NO and EDHF vasodilator mechanisms (c). (*P < 0.05, GLM-RM).

Phenylephrine (1 *μ*mol/L) caused similar contractile responses in all groups (data not shown). The responses of aortic rings to endothelium independent vasodilator SNP were not changed between groups (data not shown). Responses to the endothelium-dependent vasodilator methacholine were unchanged in saline-treated MI animals when compared with SHAM (data not shown). However, both doses of cAng (1–7) showed increased responsiveness to methacholine when compared to saline-treated MI group, which was most pronounced and only significant in the higher dose (Figure 3(a)). After blocking the NO production of endothelium with L-NAME, the response to methacholine was greatly suppressed in all the groups, however the increased responsivity of high-dose cAng (1–7) treated animals remained present (Figure 3(b)). After blocking both NO and EDHF, leaving prostaglandins as the remaining dilator factor, the difference between saline and cAng-(1–7)-treated animals disappeared (Figure 3(c)) indicating that cAng works via EDHF.

## 4. Discussion

Stimulation of the Ang-(1–7)/Mas receptor axis is a promising therapeutic strategy for treatment of MI and prevention of heart failure. For this purpose we tested the effect of the metabolically protected and Mas receptor-specific compound cAng-(1–7). Given at doses that were, respectively, 10 and 100 times lower than the minimally effective doses of native Ang-(1–7) [11], cAng-(1–7) dose-dependently lowered left ventricular weight and diastolic pressure in an MI model in which no contractile failure had yet occurred. The effect on cardiac weight seemed to depend at least partially on reduction of cardiomyocyte hypertrophy, as evidenced by the decrease in myocyte dimensions. The effects on the heart morphology and function were independent from the presence of an infarction since they also occurred in sham animals. In addition to effects on the heart, cAng-(1–7) improved peripheral endothelium-dependent vasodilation, as measured in isolated aortic rings; an effect that predominantly involved EDHF. cAng-(1–7) therefore shows favorable characteristic with regard to improvement of cardiovascular function after MI.

The present results with respect to cardiac improvement are in accordance with previous results in the MI model obtained after infusion of native Ang-(1–7) [4, 5]. A limitation of the present study, however, is the fact that infarct sizes were relatively small as compared to the previous studies, thus not allowing us to study possible beneficial effects of the compound on systolic function and cardiac fibrosis [4, 12, 13]. Nevertheless, the implications of the present study are relevant since patient populations also comprise subjects with relatively small infarct sizes but who will eventually develop heart failure, albeit after a relatively longer period. The full potential of cAng-(1–7) as an experimental drug can be appreciated from evaluation in a model of heart failure or cardiac fibrosis. The present data warrant such studies.

Endothelial dysfunction is an important hallmark in heart failure caused by MI and is believed to be pivotal in malignant cardiac remodeling due to increased afterload. Ang-(1–7) was shown to restore endothelium-dependent vasodilator function in heart failure, after stent placement, after a high salt diet and in the atherosclerosis-prone ApoE knockout mouse when infused chronically [4, 11, 14, 15]. Vascular upregulation of ACE2, which increases Ang-(1–7) levels, improves endothelial function in hypertensive rats [16]. Conversely, Mas receptor knockout or chronic treatment with A779, an antagonist of Mas receptor-associated effect diminishes endothelial function [17–19]. In accordance with the suggested role of Mas receptor signaling in improvement of endothelial function cAng-(1–7) infusion led to improved endothelial function in our rats with small MI. The improvement that was observed by us appears to be mainly caused by an increase of endothelium-derived hyperpolarizing factor (EDHF), and not through prostaglandin release. In a previous study, which involved relatively older rats that developed endothelial dysfunction after stent placement, chronic infusion of native Ang-(1–7) mainly increased prostaglandin [20]. Furthermore, short-term infusion of the native peptide improves the hypotensive response to acetylcholine through NO signaling, whilst Mas receptor knockout results in impaired NO bioavailability [19, 21]. Thus, the model that is used for studying the effect of Ang-(1–7) mediated seems to determine the signaling pathway that is improved. Our present results are to our knowledge the first to show an increased contribution of EDHF and emphasize the versatility of the therapeutic potential of the Ang-(1–7)/Mas receptor axis towards endothelial function. 

As noted above, cAng-(1–7) was intravenously administered by osmotic minipump in a dose that was 10 to 100 times lower than in previous studies the lowest efficacious dose for native Ang-(1–7). This approach allowed us to make comparisons with these previous studies and indicate that the pharmacological properties of cAng-(1–7) seem to be superior to those of native Ang-(1–7). To provide conclusive evidence it will be necessary to test cAng-(1–7) in a model of heart failure. Furthermore, a clinically relevant method of drug delivery will have to be developed. Most commonly, clinically applicable peptides are administered subcutaneously where the peptide is not degraded and which allows manipulation of the rate of peptide release, such as in the case of insulin formulations. In a recent study it was shown that subcutaneous cAng-(1–7) resulted in a 98% bioavailability. Although less efficient, oral and especially pulmonary delivery (28% bioavailability) of cAng-(1–7) appeared possible too. Therefore translation to the clinic is feasible [22]. There are other approaches to design a clinically relevant delivery method to exploit the Ang-(1–7)/Mas axis. These designs fall into four main categories: local delivery of the native peptide, nonpeptide analogues, protective incapsulation of the native peptide, and upregulation of the Ang-(1–7)-synthesizing enzyme ACE2. Local delivery is an elegant way to circumvent loss of bioavailability of Ang-(1–7). This approach has been explored to counteract problems that are associated with stent placement and has led to prevention of endothelial dysfunction [23]. Theoretically, this strategy should also be applicable for solid tumors. Peptide incapsulation includes PEG-liposome complexes that can be delivered intravenously [23], but most promising appears to be the use of hydroxypropyl *β*-cyclodextrin, which has led to successful cardioprotection after infarction or chronic isoproterenol infusion in rats when delivered orally [24]. Nonpeptide analogues include AVE 0991 and CGEN-856S, which show vasodilatory and cardioprotective properties (less arrythmias during recovery from I/R) in vitro, and antihypertensive effects in vivo [23, 25]. However, oral delivery has not been attempted with these compounds. Last, upregulation of ACE2 has been successfully attempted as intervention in cardiac and pulmonary fibrosis models, and in Ang-II-dependent renal fibrosis. Of particular interest is the use of 1-[(2-dimethylamino) ethylamino]-4-(hydroxymethyl)-7-[(4-methylphenyl) sulfonyl oxy]-9H-xanthene-9-one (XNT), an ACE2 ligand and activator of the enzyme. Until present, XNT was shown effective against cardiac and pulmonary fibrosis and against pulmonary hypertension when administered subcutaneously with minipumps [26, 27].

In summary, we here present the first data showing that lanthionine-bridged Ang-(1–7), shortly cAng-(1–7), holds promise as a therapeutic agent after MI, as it improves cardiac remodeling and endothelial function and since it has previously [22] been demonstrated that it can be delivered orally and pulmonarily. Our present results warrant further testing of this compound in various models of heart failure and possible other diseases that can be a target of beneficial Ang-(1–7)/Mas receptor axis signaling.

## Figures and Tables

**Table 1:** Weight, basic histological and cardiac parameters.

	Sham	MI	Sham	MI	MI
	saline	saline	high cAng-(1–7)	low cAng-(1–7)	high cAng-(1–7)
BW, g	477.1 ± 6.6	475.3 ± 6.4	484.6 ± 7.4	458.6 ± 10	492.3 ± 9.5
Infarct size, %	0 ± 0	15.853 ± 3.17	0 ± 0	9.721 ± 2.89	14.535 ± 4.10
Fibrosis, %	3.896 ± 0.57	3.992 ± 0.39	4.289 ± 0.54	4.695 ± 0.65	4.479 ± 0.34
HR beats/min	255.12 ± 16.0	260.3 ± 5.5	240.4 ± 9.8	274.7 ± 6.2	266.7 ± 6.2
MaxP, mmHg	107.8 ± 3.8	103.5 ± 2.9	98.6 ± 8.3	93.0 ± 4.7	96.4 ± 4.7
ESPress, mmHg	103.6 ± 4.2	100.1 ± 3.0	93.5 ± 9.3	89.4 ± 5.3	92.4 ± 5.1
dpdtMax, mmHg/sec	5572.0 ± 188.2	5456.8 ± 198.7	5533.0 ± 530.7	5026 ± 269.3	5111.3 ± 345.1
dpdtMin, mmHg/sec	−5189.1 ± 178.8	−4919.1 ± 265.2	−6123.5 ± 1268.9	−4615.4 ± 361.8	−4742.6 ± 404.9
*N*	8	25	5	12	12
